# P-370. Real-World Data on Fostemsavir in People Living with HIV: A Retrospective Chart Review

**DOI:** 10.1093/ofid/ofaf695.588

**Published:** 2026-01-11

**Authors:** Monique A Prince, Maria Akiki, Christopher S Gilbert, Jihad Slim

**Affiliations:** UCLA, Los Angeles, CA; University of Connecticut, Hartford, CT; St. George's University School of Medicine, Saint George, Saint George, Grenada; Saint Michael’s Medical Center, Newark, NJ, USA, Newark, NJ

## Abstract

**Background:**

Patients with multidrug-resistant HIV (Human Immunodeficiency Virus) face limited treatment options, increasing their risk of virologic failure. Fostemsavir, a novel attachment inhibitor, has shown efficacy and safety in clinical trials, but real-world data is lacking. We aimed to evaluate virologic suppression, immunologic response, and safety outcomes in treatment-experienced individuals in a real-world clinical setting.

**Figure:**
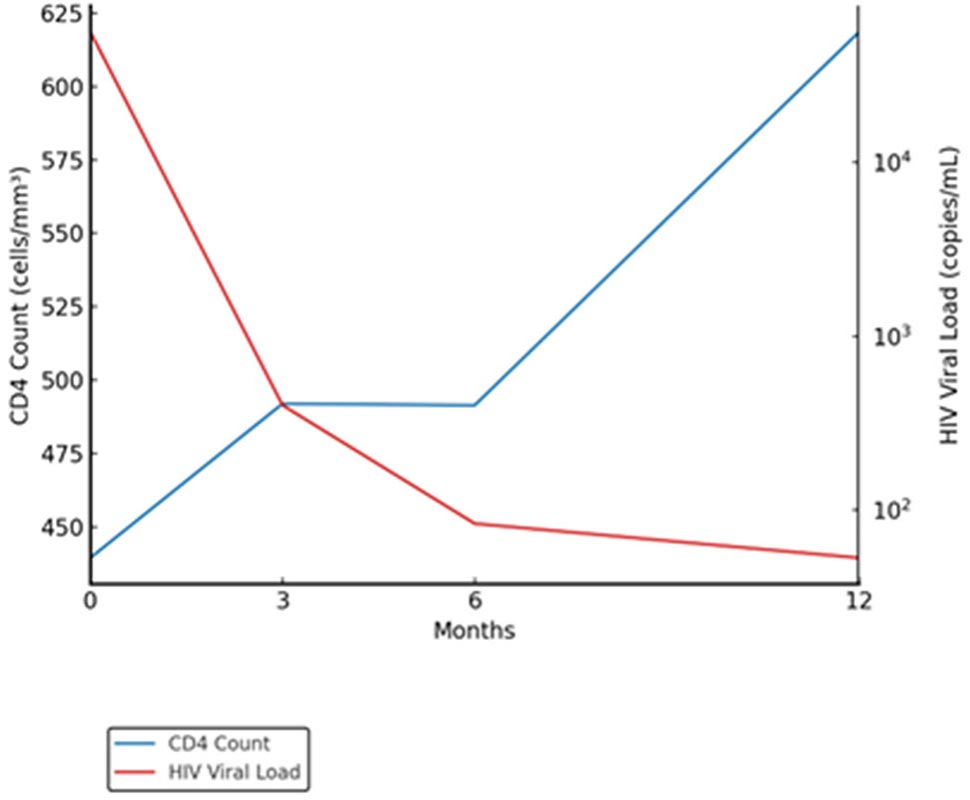
Trends in CD4 Count and HIV Viral Load Over 12 Months in Patients Receiving Fostemsavir

**Methods:**

We conducted a retrospective chart review of patients living with HIV >18 years old in a Ryan White funded HIV clinic in Newark , NJ who were treated with Fostemsavir for more than 6 months. Data on viral load, CD4 counts, adverse effects, and adherence were collected and descriptive statistics was used to analyze outcomes over 12 months. Primary outcomes were virologic suppression (HIV RNA < 20 copies/mL) and CD4 count change from baseline. Secondary outcomes assessed adherence, adverse effects, and reasons for discontinuation.

**Results:**

17 patients met the inclusion criteria, with a mean age of 57.2 years. The majority were male gender (70.6%). 58.8% were Black or African American, 17.6% White, and 23.5% identified as other races. At baseline, the mean viral load was 56,402 copies/mL, which declined to 400 copies/mL at month 3, 84 copies/mL at month 6, and 53 copies/mL at month 12. Mean CD4 count increased from 439 cells/mm³ at baseline to 492 cells/mm³ at month 3, remained stable at 491 cells/mm³ at month 6, and rose further to 619 cells/mm³ by month 12 as shown in Figure 1. Virologic suppression was achieved in most patients by month 12. Adherence was high with 82.4% fully compliant, 11.8% intermittently compliant, and 5.9% non-compliant. Adverse effects were minimal; 76% reported none, while 11.8% experienced muscle wasting, and 5.9% each reported insomnia or arthralgia. Fostemsavir was discontinued in two patients: due to virologic failure (5.9%), oral intolerance (5.9%).

**Conclusion:**

Fostemsavir demonstrated effective virologic suppression and immune recovery, with a favorable safety profile, in a real-world cohort of treatment-experienced HIV patients. These findings support its clinical utility. Ongoing research in larger populations will build on these findings to further characterize long-term outcomes.

**Disclosures:**

Jihad Slim, MD, FACP, gilead: Honoraria|merck: Honoraria|Thera: Honoraria|ViiV: Honoraria

